# Assessment of salivary thioredoxin levels in oral lichen planus and oral squamous cell carcinoma

**DOI:** 10.1002/cre2.364

**Published:** 2020-12-01

**Authors:** Maryam Amirchaghmaghi, Roya Mahfoozi, Zohreh Dalirsani, Leila Vazifeh Mostaan, Seyed Isaac Hashemy, Mohammad Taghi Shakeri

**Affiliations:** ^1^ Oral and Maxillofacial Diseases Research Center Mashhad University of Medical Sciences Mashhad Iran; ^2^ General Dentist Mashhad Iran; ^3^ Cancer Research Center Mashhad University of Medical Sciences Mashhad Iran; ^4^ Surgical Oncology Research Center Mashhad University of Medical Sciences Mashhad Iran; ^5^ Social Determinants of Health Research Center Mashhad University of Medical Sciences Mashhad Iran

**Keywords:** carcinoma, squamous cell, lichen planus, oral, saliva, squamous cell, thioredoxins

## Abstract

**Objective:**

Oral lichen planus (OLP) is a chronic mucocutaneous inflammatory disease, which is considered as a potentially malignant condition and could transformed into oral squamous cell carcinoma (OSCC). Squamous cell carcinmoma is the most common oral cancer. This study aimed to compare salivary thioredoxin levels as an antioxidant protein among patients with OSSC, OLP and healthy subjects.

**Materials and methods:**

Twenty‐eight patients with OLP, 20 patients with OSCC and 40 healthy people enrolled in this observational study. Saliva samples were collected from all subjects and salivary thioredoxin levels were evaluated by Elisa test. The data were recorded in the check lists and analyzed using SPSS (ver.17).

**Results:**

Thioredoxin levels of healthy controls were insignificantly higher than OLP and SCC patients (*p* = 0.135). There was a statistically remarkable indirect relationship between thioredoxin levels and severity of the lesions determined by Thongprasom criteria among OLP patients. The thioredoxin concentration was significantly higher in the keratotic OLP. Among the OSCC patients, the highest levels of thioredoxin were found among patients aged more than 65 years. Salivary thioredoxin levels based on other variables were not significantly different between the studied groups.

**Conclusion:**

In this study, there was not any significant difference between salivary thioredoxin levels in the OLP and OSCC patients, though it was unremarkable higher in the healthy group compared to the patients; therefore, the role of thioredoxin in the cellular oxidation–reduction status could be suggested; however, further studies are recommended.

## 1 INTRODUCTION

Lichen planus is a relatively common chronic inflammatory mucocutaneous disease with an immunologic origin. Most patients are middle‐aged adults (30–70 years), and this disease is more common in women with 3:2 ratio (M. Amirchaghmaghi, Pakfetrat, et al., 2016; Carrozzo & Thorpe, 2009). The articles reported the prevalence rates ranging from 0.5% to 2.2% for oral lichen planus (M. Amirchaghmaghi, Pakfetrat, et al., 2016), oral lichen planus (OLP) lesions are considered as a potentially malignant condition; the rate of these malignant transformation has been reported to be 0.2%–5.3% (Carrozzo & Thorpe, 2009); however, dysplastic changes even in 10% of samples were reported (Cawson, 1968).

The etiology of lichen planus is not known. Research results suggests that identifying a specific etiological factor for this condition is difficult, though it seems that the immune system plays a significant role in the incidence of this disease. T lymphocytes, which function against the epithelium, have been propounded as the first important factor for the development of OLP. Also, other factors such as inflammatory cytokines, imbalance of antioxidants are also involved in the pathogenicity of disease (Ismail et al., 2007). Nevertheless, the role of factors such as stress, local trauma, infection, and lifestyle cannot be neglected (Ismail et al., 2007).

Oxidative stress leads to impair in the balance between oxidants and antioxidants, resulting in increased oxidative damage to lipids, DNA molecules and proteins in patients with OLP and could act as a trigger in the initiation or progression of many diseases (Ergun et al., 2011). Among mucosal disorders, the role of oxidative stress has been proven in some diseases including psoriasis, lichen planus, and pemphigus vulgaris (Yesilova et al., 2013). Since the human body is constantly exposed to different endogenous and exogenous reactive oxygen, it is equipped with any non‐enzymatic and enzymatic antioxidants such as vitamin C, vitamin E, catalase, super oxidase dismutase, and many others that are essential for the human health and cell survival (Kurutas, 2015). The thioredoxin is one of the major oxidoreductases of cells composed of thioredoxin‐thioredoxin reductase system, and NADPH. The role of this system has been discussed extensively in the health and disease (Kiafar et al., 2020; Sobhani et al., 2016; Yaghoubi et al., 2019).

SCC is a multifactorial disease; no single etiological factor has been clearly defined or accepted. It is possible that more than one factor is required for development of this malignancy (co‐carcinogenesis) (Kumar et al., 2016). Human thioredoxin system plays a significant role in establishing the homeostasis of cellular oxidation–reduction status. In some studies, a paradoxical role has been discussed for thioredoxin; it prevents cancer; on the other hand, it acts in its development. This system prevents cancer through removing carcinogen antioxidants and repairing mutated DNA. On the other side, it has been found that thioredoxin is involved in the growth of cancer cells. In addition, redox system is associated with tumor angiogenesis through inducing vascular endothelial growth factor. Thioredoxin‐thioredoxin reductase system plays a key role in development of cancer cells. Some studies have also suggested that this system plays a significant role in the growth of tumors and resistance against some chemotherapeutic agents in many malignancies (Iwasawa et al., 2011; Mohammadi et al., 2019).

Although the role of thioredoxin system in the proliferation of tumoral cells and resistance to chemotherapeutic drugs has been found in some neoplasms, its role in oral SCC has remained unclear. Only some limited studies including Iwasawa et al. have reported elevated thioredoxin reductase in OSCC, whereby they found it to be related to metastasis of tumor to the lymph nodes (Sobhani et al., 2016).

Considering the mechanisms mentioned for thioredoxin in SCC, as well as; its possible role in lichen planus disease, this research was performed to assess the salivary antioxidant levels through measuring thioredoxin in patients with oral SCC, as a malignant lesion, and compare with OLP patients, as a premalignant lesion, and healthy individuals.

## 2 MATERIALS AND METHODS

### 2.1 Study groups characteristics

This study was performed as an observational study during October 2018–August 2019, performed on 40 healthy individuals, 28 patients with OLP (both erosive and non‐erosive), and 20 patients with OSCC (oral cavity SCC) referring to the oral and maxillofacial medicine department of Mashhad Faculty of Dentistry, Shahab Density Clinic, and Omid Hospital in Mashhad. The patients with lichen planus, which were confirmed through clinical and histopathological investigations, according to WHO modified criteria (Thongprasom et al., 1992) and met other inclusion criteria, enrolled in this study. Diagnosis of SCC had been confirmed through histopathological examination. The healthy individuals were selected from the patients referring to the oral and maxillofacial medicine department of Mashhad Faculty of Dentistry, who had no special lesion or systemic disease. Among the three subject groups, there were no significant differences in terms of mean age, gender or smoking history. The inclusion criteria for oral SCC and oral lichen planus were as follows: (1) patients with lichen planus lesions or OSCC as confirmed by clinical and histopathological examination (new case) (2) patients who had signed the informed consent form. The exclusion criteria were as follows: (1) the subjects (OLP, SCC or healthy groups), who had used vitamin supplements and patients with previous malignancies or systemic comorbidities (2) the patients, whose histopathological result was reported as lichenoid reaction, (3) patients with OLP or OSCC, who had undergone treatment. All patients completed and signed written informed consent forms. The protocol of this study was approved by ethical committee of Mashhad University of Medical Sciences (the ethical code: (IR.MUMS.DENTISTRY.REC.1397.087).

A checklist was completed for all subjects including demographic characteristics (age, gender, and medical history), lesion characteristics (site of lesion, type and severity of lesion for OLP patients based on the Thongprasom criteria (Thongprasom et al., 1992), clinical features: (OLP: keratotic and non‐keratotic; and SCC: ulcer, white plaques, red plaques, and exophytic lesion).

### 2.2 Experimental methods for assessing thioredoxin

For all of the three groups, the patients with OSCC, OLP, and healthy individuals, saliva sampling was performed. The subjects were recommended not to drinking, eating, or smoking for 90 min before collecting unstimulated saliva through spitting method. Saliva samples were collected between 9 a.m. and 11 a.m. The patients were asked to sit in a convenient position and gather the saliva in their mouth and then spit it in a falcon sterilized tube. This was performed every 60 s for 5–15 min. Saliva samples were kept on ice and sent to the laboratory for processing (Shirzaiy et al., 2017). Until the time of investigation, the samples were kept at −20°C. In order to separate squamous cells and debris and mucous, the saliva samples were centrifuged immediately after the de‐frizzing for 15 min (3000 g at 25°C). We placed the samples in the test tubes with the volume of 10 cc. Next, in order for the mucin present in the saliva to be lysed (mucolysis), the samples were placed inside a fridge for 24 h at −20°C. After 24 h, the samples were centrifuged (3000 g for 10 min). Eventually, the watery part of the saliva was transferred to micro tubes with a volume of 1.5 cc. Next, the level of thioredoxin in the watery part of saliva was measured by the kits of ZellBio Co., Germany (Human TRX ELISA kit with the product code of ZB‐11452) and based on the protocol presented by the kit manufacturer's company.

First, the reagents, samples, and standards were prepared. Then, 40 μl of the sample(s) and 10 μl of thioredoxin antibody were added to 50 μl of standard and 50 μl of streptavidin‐HPR, and placed at 37°C for 60 min. Next, the containers were washed five times with 300 μl diluted buffer. Thereafter, 50 μl of chromogen A solution and 50 μl of chromogen B solution were added and placed at 37°C for 10 min for color detection until incubation. Then, 50 μl of stop solution was added. Finally, the optical density (OD) was measured at the wavelength of 450 nm.

### 2.3 Statistical analysis

The information collected for each patient was introduced into a special form and then statistical analysis was performed. The data was entered into SPSS software (version 17) for statistical analysis. Kolmogorov–Smirnov normality test was used for assessment of normal distribution of the quantitative variables. In order to compare and assess the qualitative variables between the groups, Chi‐do and Fisher's exact test were employed. Parametric statistical methods (independent *t*‐test, analysis of variance [ANOVA]), for quantitative variables in case of normal distribution, and nonparametric methods (Mann–Whitney test, Kruskal‐Wallis) for abnormal distribution, were used. The significance level in the statistical tests was considered as 0.05.

## 3 RESULTS

In this research, 28, 20, and 40 patients with OLP (31.8%), with OSCC (22.7%), and healthy individuals (45.5%), respectively, who met the inclusion criteria were included. These individuals consisted of 45 females (51.1%) and 43 males (48.9%). Their mean age was 50.40 ± 12.31 with the age range of 25–88 years. The mean age of women and men were 48.47 ± 10.881 and 52.42 ± 13.489 years. The demographic characteristics of the patients for each study group are mentioned in Table 1. The individuals were evaluated in terms of the salivary thioredoxin levels and its association with other factors such as age, gender, clinical features, severity and site of lesions.

**TABLE 1:** Information of studied subjects

		OLP	OSCC	Control	*p* value
		*N* (%)	*N* (%)	*N* (%)	
Age (years)					0.001
25–45		13 (46)	3 (15)	17 (42.5)	
46–65		15 (54)	10 (50)	22 (55)	
65<		0	7 (35)	1 (2.5)	
Gender					0.005
Female		21 (75)	6 (30)	18 (45)	
Male		7 (25)	14 (70)	22 (55)	
Location					
Lip		9 (14.06)	3 (15)		
Buccal mucosa		19 (29.68)	4 (20)		
Gingiva		16 (25)	3 (15)		
Tongue		15 (23.43)	9 (45)		
Retromolar pad		2 (3.125)	1 (5)		
Floor of mouth		1 (1.56)	0		
Palate		2 (3.125)	0		
Clinical features					
OLP	Keratotic	7 (25)			
	Eutrophic	10 (35.7)			
	Erosive	11 (39.3)			
SCC	Ulcerative	7 (35)			
	Leukoplakia	10 (50)			
	Erythroplakia	1 (5)			
	Exophytic	2 (10)			
Severity of OLP lesions (Thongprasom criteria)					
1		7 (25)			
2		5 (11.8)			
3		5 (17.8)			
4		6 (21.6)			
5		7 (17.8)			
Thioredoxin (unit/L)		OLP	SCC	Control	*p* value
		112.28 ± 982.0491	122.25 ± 850.1752	125.19 ± 988.6790	0.135

**FIGURE 1:**
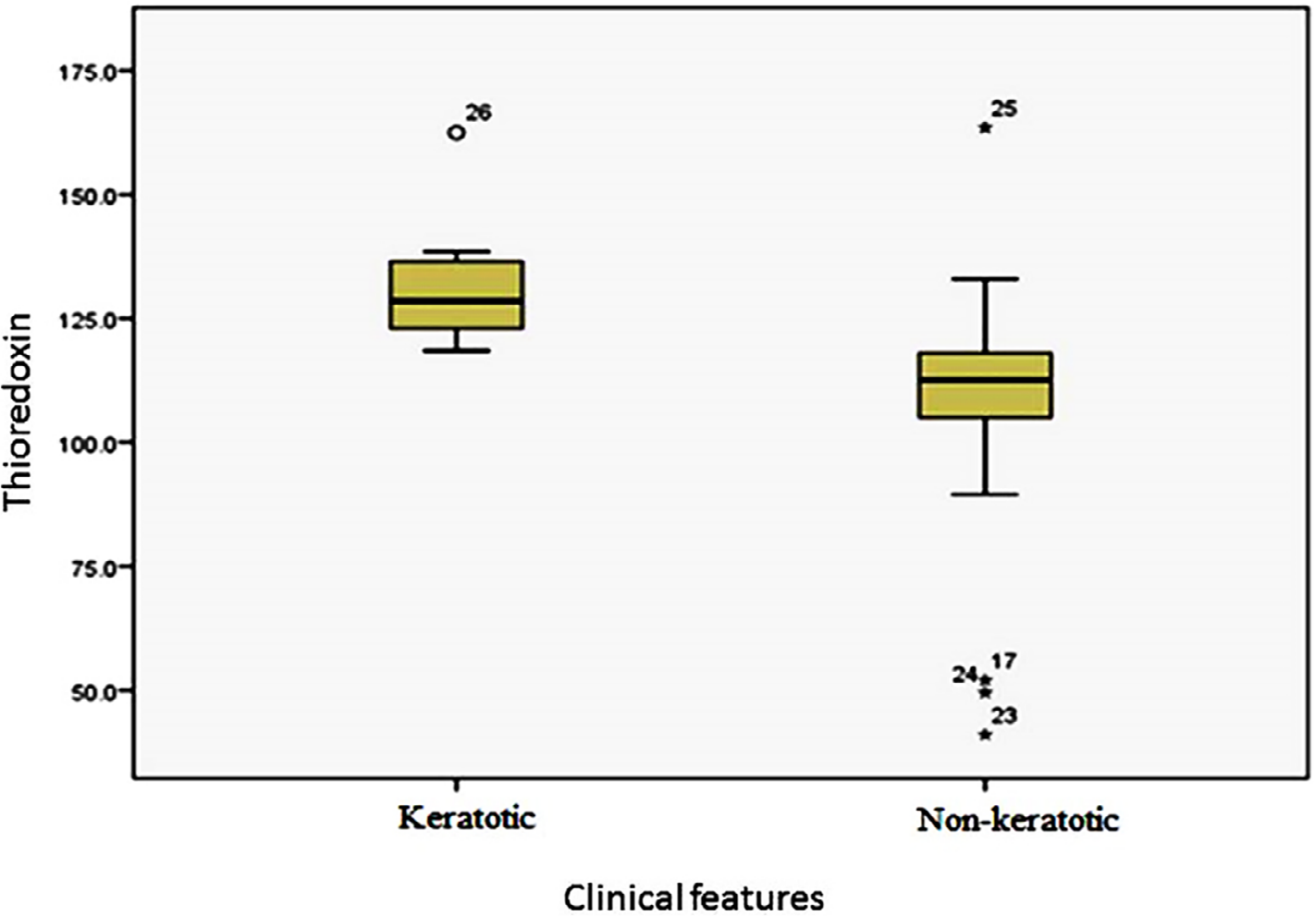
Box plot of thioredoxin levels (unit/L) in the OLP subjects according to clinical features

The levels of thioredoxin was measured in OLP and OSCC and healthy individuals groups and there was not any significant different between them (*p* = 0.135), though it was higher in the healthy subjects as compared to other groups (Table 2). The levels of thioredoxin did not differ significantly between the healthy and OLP subjects in terms of age and gender. However, thioredoxin levels in patients with OSCC, with the age above 65 years were significantly higher than other ones (gender: *p* = 0.130; age: *p* = 0.001) (Table 1). Furthermore, investigation of the thioredoxin levels in terms of the clinical features of lesions indicated that in keratotic OLP patients, thioredoxin levels were significantly higher than the non‐keratotic features (*p* = 0.003) (Figure 1).

**TABLE 2:** The levels of thioredoxin in the OLP and OSCC and healthy individuals groups

	Subjects	Number	Mean ± SD thioredoxin (unit/L)	*p* value^a^
Groups	OLP	28	112.982 ± 28.0497	0.135
	SCC	20	122.850 ± 25.1752	
	Normal	40	125.988 ± 19.6790	

^a^ Kruskal‐Wallis test.

In the patients with OLP, when the severity of lesions increased, thioredoxin levels decreased, and this difference was only significant between severities of 1 and 3 (in terms of Thongprasom criteria) (*p* = 0.013) (Table 3).

**TABLE 3:** The levels of thioredoxiin the OLP patients according to Thongprasom criteria

	Severity of lesions (Thongprasom)	Number	Mean ± SD thioredoxin (unit/L)	*p* value^a^
OLP	1	7	132.643 ± 14.9268	0.013
	2	5	126.200 ± 21.7675	
	3	5	97.000 ± 26.0000	
	4	6	99.833 ± 32.2053	
	5	5	104.000 ± 30.9213	
	Total	28	112.982 ± 28.0497	

^a^ Kruskal‐Wallis test.

**FIGURE 2:**
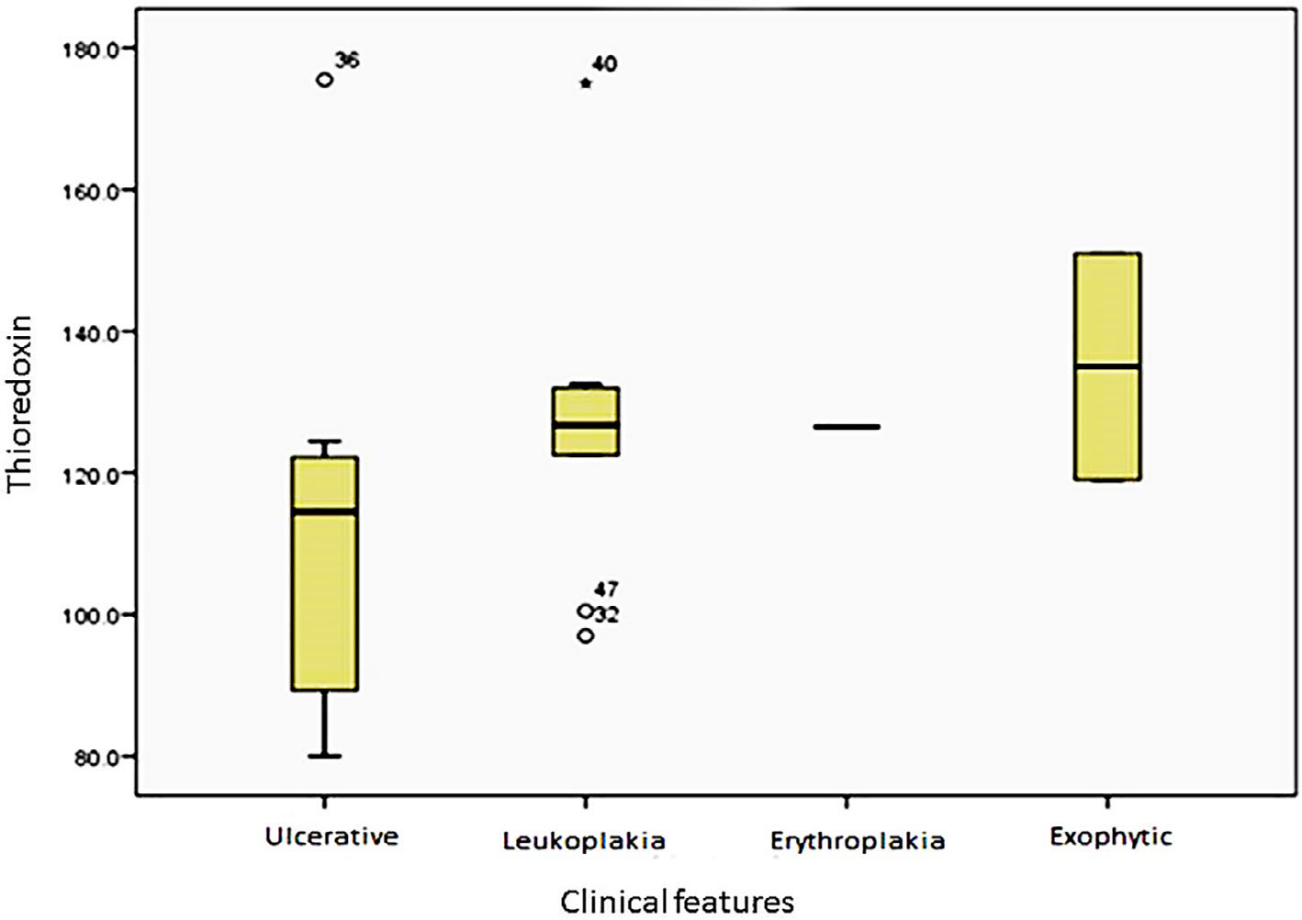
Box plot of thioredoxin levels (unit/L) in the SCC subjects according to clinical features

In patients with OSCC, the thioredoxin levels in the exophytic type were insignificantly greater than other presentations (*p* = 0.383) (Figure 2).

Histopathological assessment of SCC lesions revealed that 12 patients (80%) had grade I, and 3 patients (20%) had grade II of tumor; however, there was not any remarkable correlation between tumor grade and salivary thioredoxin levels (*p* = 0.219).

Finally, thioredoxin levels did not differ significantly in terms of site of lesion (OLP: *p* = 0.156; SCC: *p* = 0.351).

## 4 DISCUSSION

Oral cancer is a complex and multidimensional process, and in most cases it begins with epithelial dysplasia. Nevertheless, effective interventional measures for treating or delaying conversion of potentially malignant to malignant lesions are limited. Specific rise of oxidative stress levels in cancerous cells is a result of an imbalance between production and removal of reactive oxygen species (ROS). It has been reported that oxidative stress contributes to transformation of dysplasia to oral cancer. Human thiordoxin‐1 (Trx‐1) belongs to a family of small redox proteins reduced by Trx reductase (TrxR) and NADPH. Trx‐1 plays a significant role in maintaining redox balance, which is essential for cell survival, tumor growth, and angiogenesis (Chen et al., 2018; S. I. Hashemy, 2011).

A study was performed by Zhu et al. to investigate Trx‐1 and Trx‐1 reductase in the histopathological samples of patients with lingual squamous cell carcinoma. Elevation of Trx‐1 in this study had a significant relationship with the degree of differentiation of tumoral cells and five‐year survival of patients. In this study, it was found that Trx‐1 elevation predicts a poor prognosis for patients with lingual SCC (Zhu et al., 2011).

In other study, HU et al. used matrix‐assisted laser desorption/ionization – mass spectrometry (MALDI‐TOF MS) analysis to compare oral fluid samples from 20 oral cancer and 20 control subjects and indicated that the mean level of thioredoxin in cancer samples was higher than normal samples (Hu et al., 2007). In our study, Trx‐1 levels were compared between SCC and healthy subjects. Unlike the study by Zhu and HU, Trx levels were found to be lower in SCC patients than in healthy individuals. Further, in the present study, which was performed on saliva samples unlike the research by Zhu, it did not have any significant relationship with clinical features, gender, and site of tumor. Since two different roles have been propounded for the thioredoxin system in carcinogenesis pathway, it seems that in the study by Zhu et al., Trx is involved in stimulating cell growth and increased growth of the tumor. This role has also been confirmed for other cancers including lung, thyroid, breast, and colorectal; (Zhu et al., 2011), In our study, concerning the lower level of Trx in patients with SCC and OLP in comparison to healthy individuals, another theory can be put forward for the role of Trx, as explained in the review study by Shabani et al., which mentioned a cancer preventive role for Trx. As it could play a role in protecting cell DNA against mutation and oxidative stress damages and might reduce incidence of malignant changes. Moreover, Shabani et al., indicated another role of Trx in stimulating the growth of tumoral cells and angiogenesis, as also reported in some other studies. It has been stated that both roles of Trx system require further investigations (Shabani et al., 2014).

The studies by Benhar et al. as well as Harris et al. suggested that thioredoxin and glutaredoxin, which are important players for the redox system, have powerful and synergistic antitumor effects. Also, in these studies, an important role has been propounded for thioredoxin in ROS removal and maintaining cellular homeostasis (Benhar et al., 2016; Harris et al., 2015).

The study by Calderia et al. on the histopathological samples of healthy individuals, actinic cheilitis, and lip SCC, revealed that the rate of expression of thioredoxin was higher in most cancer cells. In this study, thioredoxin levels were higher in actinic cheilitis than in SCC of the lip; since UV radiation can influence the expression of thioredoxin, this overexpression of thioredoxin can be associated with the antioxidant role of this system. Nevertheless, some malignant cells had low or unmeasurable thioredoxin levels; possibly in these cells, glutaredoxin is involved in DNA synthesis (Caldeira et al., 2017). In our study, in OLP patients, thioredoxin levels were higher in lip lesions, while in SCC samples the minimum level of thioredoxin was observed in the lip involvement. Nevertheless, no significant difference was observed between thioredoxin levels in the different sites of lesions either in OLP or OSCC. Also, elevated Trx‐1 levels were assessed in the elderly patients, which might be related to poor prognosis of SCC in the older subjects; concurrently, Zhu et al. showed increased thioredoxin predicts a poor prognosis for SCC patients (Zhu et al., 2011). Though there is not any information in the literature concerning the changes of thioredoxin levels in elderly, it seems that the redox status shifts in favor of oxidation through ageing, and this oxidative stress may lead to the overexpression of antioxidants such as thioredoxin to maintain the redox balance.

In a study by Chen et al., both human and animal tissue samples were investigated. Based on the results, among the different proteins involved in dysplasia, although Trx‐1 showed the maximum impact on dysplasia stages in the human and animal and other proteins including gluthardoxin‐1 and proxyredoxin‐2, were also effective in this process (Chen et al., 2018). In our study, it was found that thioredoxin levels did not differ significantly across the healthy, OLP, and OSCC samples. However, in OLP lesions, the thioredoxin levels had grown significantly in keratotic types with a less severe clinical severity (based on Thongprasom criteria). Since in keratotic types and less intense OLP conditions, the probability of dysplasia is lower, thus higher thioredoxin levels suggests its active antioxidant and preventing role in these lesions. Also, there was not any significant difference between various clinical and pathological subtypes of OSCC patients in the present study, which could be related to few numbers of lesions with different histopathological grades of SCC.

The role of oxidative stress, as one of the etiological factors, in OLP has recently been examined in different papers. In two studies, some factors of oxidative stress and C‐reactive protein (CRP) were examined in patients with OLP and a significant rise in the serum malondialdehyde and isoprostane levels was seen in OLP patients as compared to healthy subjects (M. Amirchaghmaghi, Hashemy, et al., 2016; S. I. Hashemy et al., 2016). On the other hand, in the study by Amirchaghmaghi et al. the level of isoprostane 8 was significantly higher in the non‐keratotic OLP than in the keratotic type, which was correspondent with ours results about thioredoxin (M. Amirchaghmaghi, Hashemy, et al., 2016).

One of the strong points of this study was concurrent investigation of thioredoxin in patients with OLP, as a premalignant lesion and OSCC, as the most common malignancy of the oral cavity, as well as use of saliva as a noninvasive and convenient method for investigating thioredoxin.

There is not any information regarding the correlation between serum and salivary thioredoxin; however, thioredoxin is a small protein (12 KD) and it is expected to be found in saliva. Therefore, it seems logical to claim that any change in the blood proteins levels could reflect in the patients' saliva. Moreover, serum and salivary thioredoxin has been studied in previous studies (Soyama et al., 2020).

It is suggested to design a comprehensive study for concurrent assessment of thioredoxin reductase and thioredoxin levels and investigation of the correlation between the levels of thioredoxin and response to treatment, metastasis, and different subtypes of OSCC and dysplasia of OLP.

## 5 CONCLUSION

Based on the results of the present study, salivary thioredoxin was lower in the patients with OLP or OSCC as compared to healthy individuals; however, it is not statistically significant. Furthermore, a significant relationship was observed between thioredoxin levels and the severity of OLP based on Thongprasom criteria. Thus, it is possible that thioredoxin may be involved in the pathogenesis of these two diseases. Moreover, in patients with OSCC, thioredoxin was significantly correlated with older age, which can be considered for further detailed studies.

## CONFLICTS OF INTEREST

The authors declare that they have no conflict of interest.

## References

[cre2364-bib-0001] Amirchaghmaghi, M., Hashemy, S. I., Alirezaei, B., Keyhani, F. J., Kargozar, S., Vasigh, S., … Pakfetrat, A. (2016). Evaluation of plasma isoprostane in patients with oral lichen planus. Journal of Dentistry, 17(1), 21–25. 26966704 PMC4771048

[cre2364-bib-0002] Amirchaghmaghi, M., Pakfetrat, A., Delavarian, Z., Ghalavani, H., & Ghazi, A. (2016). Evaluation of the efficacy of curcumin in the treatment of oral lichen planus: A randomized controlled trial. Journal of Clinical and Diagnostic Research: JCDR, 10(5), ZC134–ZC137. 27437348 10.7860/JCDR/2016/16338.7870 PMC4948524

[cre2364-bib-0003] Benhar, M., Shytaj, I. L., Stamler, J. S., & Savarino, A. (2016). Dual targeting of the thioredoxin and glutathione systems in cancer and HIV. The Journal of Clinical Investigation, 126(5), 1630–1639. 27135880 10.1172/JCI85339 PMC4855928

[cre2364-bib-0004] Blasberg B, G. M (2015). Temporomandibular Disorders.

[cre2364-bib-0005] Caldeira, P. C., Silva, L. S., Batista, A. C., & Aguiar, M. C. F. (2017). Thioredoxin and metallothionein: Homeostasis‐related proteins in lip carcinogenesis. Archives of Oral Biology, 77, 75–81. 28183007 10.1016/j.archoralbio.2017.01.020

[cre2364-bib-0006] Carrozzo, M., & Thorpe, R. (2009). Oral lichen planus: A review. Minerva Stomatologica, 58(10), 519–537. 19893476

[cre2364-bib-0007] Cawson, R. (1968). Treatment of oral lichen planus with betamethasone. British Medical Journal, 1(5584), 86–89. 5634718 10.1136/bmj.1.5584.86 PMC1984707

[cre2364-bib-0008] Chen, X., Hu, Q., Wu, T., Wang, C., Xia, J., Yang, L., … Chen, X. (2018). Proteomics‐based investigation of multiple stages of OSCC development indicates that the inhibition of Trx‐1 delays oral malignant transformation. International Journal of Oncology, 52(3), 733–742. 29328386 10.3892/ijo.2018.4235 PMC5807042

[cre2364-bib-0009] Ergun, S., Troşala, Ş. C., Warnakulasuriya, S., Özel, S., Önal, A. E., Ofluoğlu, D., … Tanyeri, H. (2011). Evaluation of oxidative stress and antioxidant profile in patients with oral lichen planus. Journal of Oral Pathology & Medicine, 40(4), 286–293. 21039889 10.1111/j.1600-0714.2010.00955.x

[cre2364-bib-0010] Harris, I. S., Treloar, A. E., Inoue, S., Sasaki, M., Gorrini, C., Lee, K. C., … Cox, M. A. (2015). Glutathione and thioredoxin antioxidant pathways synergize to drive cancer initiation and progression. Cancer Cell, 27(2), 211–222. 25620030 10.1016/j.ccell.2014.11.019

[cre2364-bib-0011] Hashemy, S. I. (2011). The human thioredoxin system: Modifications and clinical applications. Iran. J. Basic Med. Sci, 14(3), 191–204.

[cre2364-bib-0012] Hashemy, S. I., Gharaei, S., Vasigh, S., Kargozar, S., Alirezaei, B., Jahed Keyhani, F., & Amirchaghmaghi, M. (2016). Oxidative stress factors and C‐reactive protein in patients with oral lichen planus before and 2 weeks after treatment. Journal of Oral Pathology & Medicine, 45(1), 35–40. 26058710 10.1111/jop.12326

[cre2364-bib-0013] Hu, S., Yu, T., Xie, Y., Yang, Y., Li, Y., Zhou, X., … Wong, D. T. (2007). Discovery of oral fluid biomarkers for human oral cancer by mass spectrometry. Cancer Genomics & Proteomics, 4(2), 55–64. 17804867

[cre2364-bib-0014] Ismail, S. B., Kumar, S. K., & Zain, R. B. (2007). Oral lichen planus and lichenoid reactions: Etiopathogenesis, diagnosis, management and malignant transformation. Journal of Oral Science, 49(2), 89–106. 17634721 10.2334/josnusd.49.89

[cre2364-bib-0015] Iwasawa, S., Yamano, Y., Takiguchi, Y., Tanzawa, H., Tatsumi, K., & Uzawa, K. (2011). Upregulation of thioredoxin reductase 1 in human oral squamous cell carcinoma. Oncology Reports, 25(3), 637–644. 21206984 10.3892/or.2010.1131

[cre2364-bib-0016] Kiafar, B., Binabaj, M. M., Jafarian, A. H., Khazan, Z., & Hashemy, S. I. (2020). The relationship between tissue thioredoxin reductase activity and the psoriasis area and severity index. Indian Journal of Dermatology, 65(1), 29–32. 32029936 10.4103/ijd.IJD_327_18 PMC6986113

[cre2364-bib-0017] Kumar, M., Nanavati, R., Modi, T. G., & Dobariya, C. (2016). Oral cancer: Etiology and risk factors: A review. Journal of Cancer Research and Therapeutics, 12(2), 458–463. 27461593 10.4103/0973-1482.186696

[cre2364-bib-0018] Kurutas, E. B. (2015). The importance of antioxidants which play the role in cellular response against oxidative/nitrosative stress: Current state. Nutrition Journal, 15(1), 71. 10.1186/s12937-016-0186-5 PMC4960740 27456681

[cre2364-bib-0019] Mohammadi, F., Soltani, A., Ghahremanloo, A., Javid, H., & Hashemy, S. I. (2019). The thioredoxin system and cancer therapy: A review. Cancer Chemotherapy and Pharmacology, 84(5), 925–935. 31367788 10.1007/s00280-019-03912-4

[cre2364-bib-0020] Shabani, S., Nourizadeh, N., & Soltankhah, M. (2014). The over expression of thioredoxin during malignancies. Reviews in Clinical Medicine, 1(4), 218–224.

[cre2364-bib-0021] Shirzaiy, M., Ladiz, M. A. R., Dalirsani, Z., Haghighi, J. D., & Nakhaii, A. (2017). Evaluation of salivary total antioxidant capacity in smokers with severe chronic periodontitis. International Journal of High Risk Behaviors and Addiction, 6(3), e59486.

[cre2364-bib-0022] Sobhani, M., Taheri, A.‐R., Jafarian, A.‐H., & Hashemy, S. I. (2016). The activity and tissue distribution of thioredoxin reductase in basal cell carcinoma. Journal of Cancer Research and Clinical Oncology, 142(11), 2303–2307. 27601162 10.1007/s00432-016-2242-0 PMC11819213

[cre2364-bib-0023] Soyama, T., Masutani, H., Hirata, C. L., Iwai‐Kanai, E., & Inamoto, T. (2020). Thioredoxin as a novel sensitive marker of biological stress response in smoking. Journal of Clinical Biochemistry and Nutrition, 67(3), 228–231. 33293762 10.3164/jcbn.19-108 PMC7705090

[cre2364-bib-0024] Thongprasom, K., Luangjarmekorn, L., Sererat, T., & Taweesap, W. (1992). Relative efficacy of fluocinolone acetonide compared with triamcinolone acetonide in treatment of oral lichen planus. Journal of Oral Pathology & Medicine, 21(10), 456–458. 1460584 10.1111/j.1600-0714.1992.tb00974.x

[cre2364-bib-0025] Yaghoubi, N., Youssefi, M., Hashemy, S. I., Rafat Panah, H., Mashkani, B. A., & Zahedi Avval, F. (2019). Thioredoxin reductase gene expression and activity among human T cell lymphotropic virus type 1–infected patients. Journal of Medical Virology, 91(5), 865–871. 30489643 10.1002/jmv.25371

[cre2364-bib-0026] Yesilova, Y., Ucmak, D., Selek, S., Dertlioğlu, S., Sula, B., Bozkus, F., & Turan, E. (2013). Oxidative stress index may play a key role in patients with pemphigus vulgaris. Journal of the European Academy of Dermatology and Venereology, 27(4), 465–467. 22324759 10.1111/j.1468-3083.2012.04463.x

[cre2364-bib-0027] Zhu, X., Huang, C., & Peng, B. (2011). Overexpression of thioredoxin system proteins predicts poor prognosis in patients with squamous cell carcinoma of the tongue. Oral Oncology, 47(7), 609–614. 21652258 10.1016/j.oraloncology.2011.05.006

